# Open total talus dislocation without concomitant malleoli fracture: a case report

**DOI:** 10.1186/s13256-024-04632-x

**Published:** 2024-08-14

**Authors:** Sameer Lamichhane, Rajiv Maharjan, Pramesh Thapa, Binit Dhakal, Amit Dhungana

**Affiliations:** 1 Dhaulagiri Hospital, Baglung, Nepal; 2 Department of Orthopaedics, BPKIHS, Dharan, Nepal; 3 Dhaulagiri Hospital, Baglung, Nepal

**Keywords:** Avascular necrosis, Dislocation, Reduction, Stabilization, Talus

## Abstract

**Background:**

Total talus dislocation without ankle (malleoli) fracture is a very rare injury with prevalence of only 0.06% of all dislocations and only 2% of talar injuries, and are usually associated with common complications such as infection, avascular necrosis, and posttraumatic arthritis. The treatment usually involves debridement, reduction, stabilization of the ankle joint, and primary or secondary closure of the wound.

**Case presentation:**

We present the case of a 40-year-old South Asian woman who was involved in an accident. She was rushed to our hospital, whereby subsequent examination revealed an open total talus dislocation with the talus being exposed in its entirety from a contaminated wound in the medial side. Furthermore, radiograph confirmed total talus dislocation without concomitant malleoli fracture. She was immediately taken to the operating theater whereby debridement and immediate reduction was performed under anesthesia, and the ankle was stabilized with external fixator for about 6 weeks. She is now able to bear weight on the affected ankle with minimal tolerable pain and has normal range of motion of the ankle.

**Conclusions:**

Open total talus dislocation without concomitant malleoli fracture is a rare injury. Reduction of the talus in combination with complete wound debridement potentially successfully avoids infection, provides early revascularization preventing avascular necrosis, and preserves the normal ankle anatomy.

## Background

The talus is the most vulnerable tarsal bone in terms of vascularity that has no muscular or tendinous connections and has most of its surface lined with articular cartilage [1]. Due to the position of the talus, in addition to its function in transferring load, the major talar injuries and their sequelae lead to significant disability [2, 3]. Total talus dislocation is when the talus is entirely detached and dissociated from all surrounding joints. Total dislocation of the talus is a rare injury and has prevalence of only 0.06% of all dislocations and only 2% of talar injuries [4]. In instances of injury, infection, avascular necrosis (AVN), instability, and posttraumatic arthritis are the most commonly anticipated complications [5]. This case report describes a patient with total talus dislocation without concomitant malleoli fracture who was treated with early debridement, reduction, and stabilization of ankle joint.

## Case presentation

**Fig.1:**
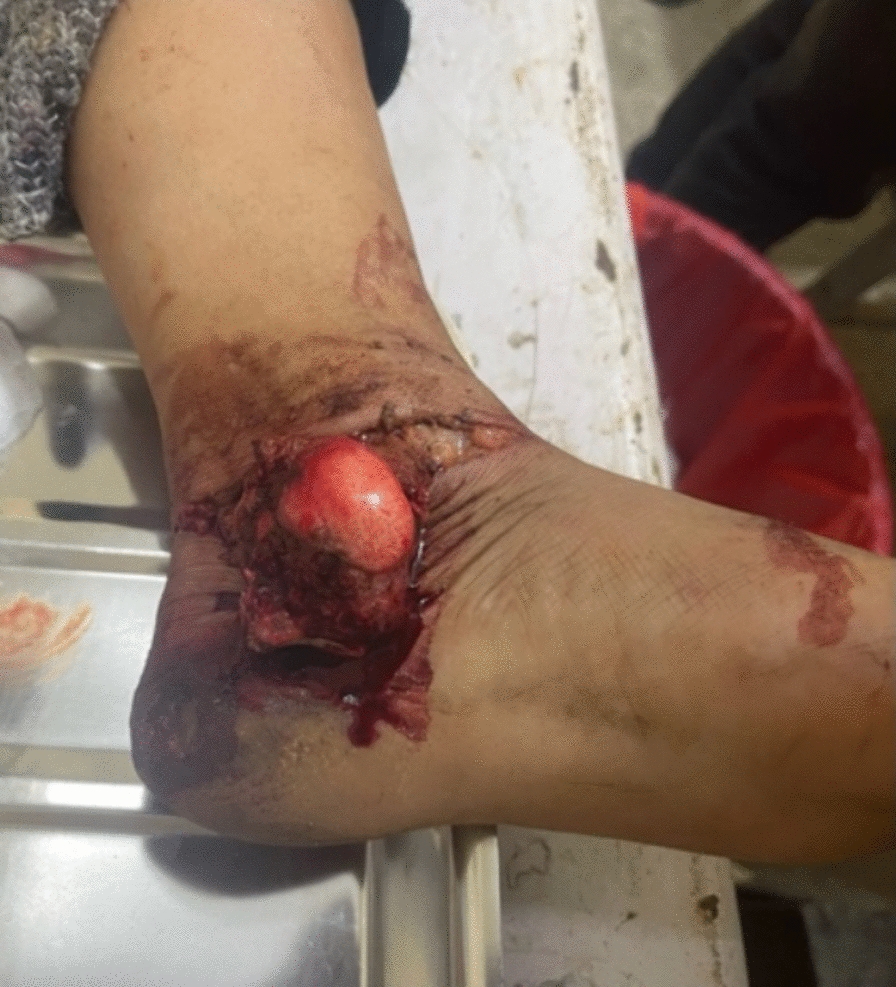
Clinical picture showing total talus dislocation

**Fig.2:**
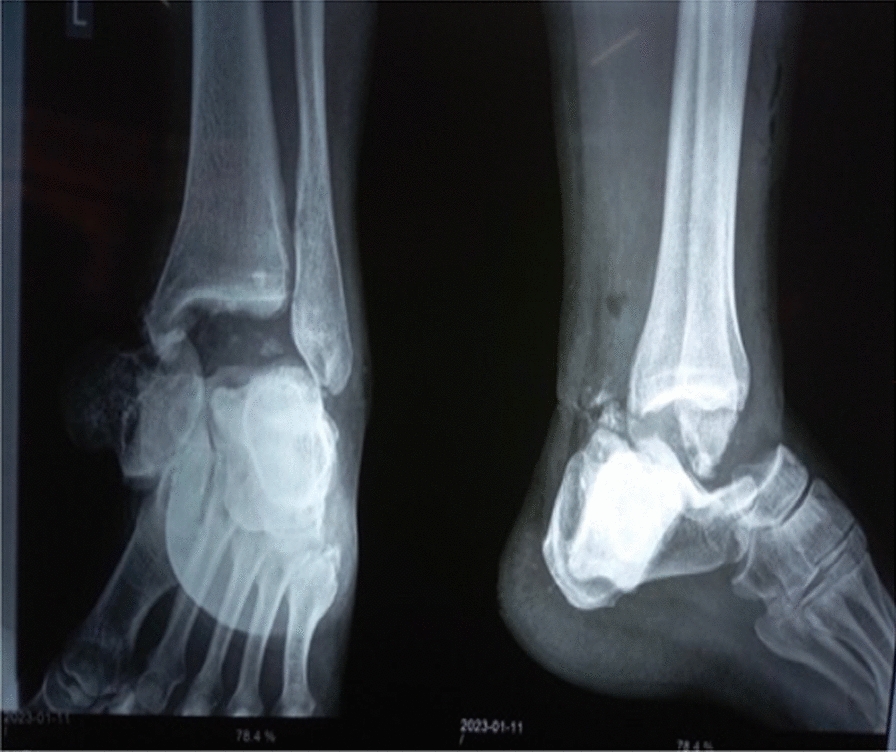
Radiograph showing total talus dislocation

A 40-year-old South Asian female, with no significant medical history, was involved in a construction accident (she fell in a pit and was buried by mud) with the point of impact over her left foot. She was taken out and rushed to the emergency of our hospital immediately within 3 hours. Physical examination disclosed a contaminated wound on the medial aspect of the ankle (Fig. 1). The talus was completely dislocated but was connected to some soft tissue. There were no other apparent injuries nor any distal neurovascular deficit. Triple antibiotics were administered 3 hours after the injury and immediate irrigation of the wound was done. Computed tomography scan was not done as it was not available in our hospital/locality and referral to another center would delay in prompt debridement; however, plain radiograph showed complete talus dislocation without concomitant malleoli fracture (Fig. 2).

**Fig.3:**
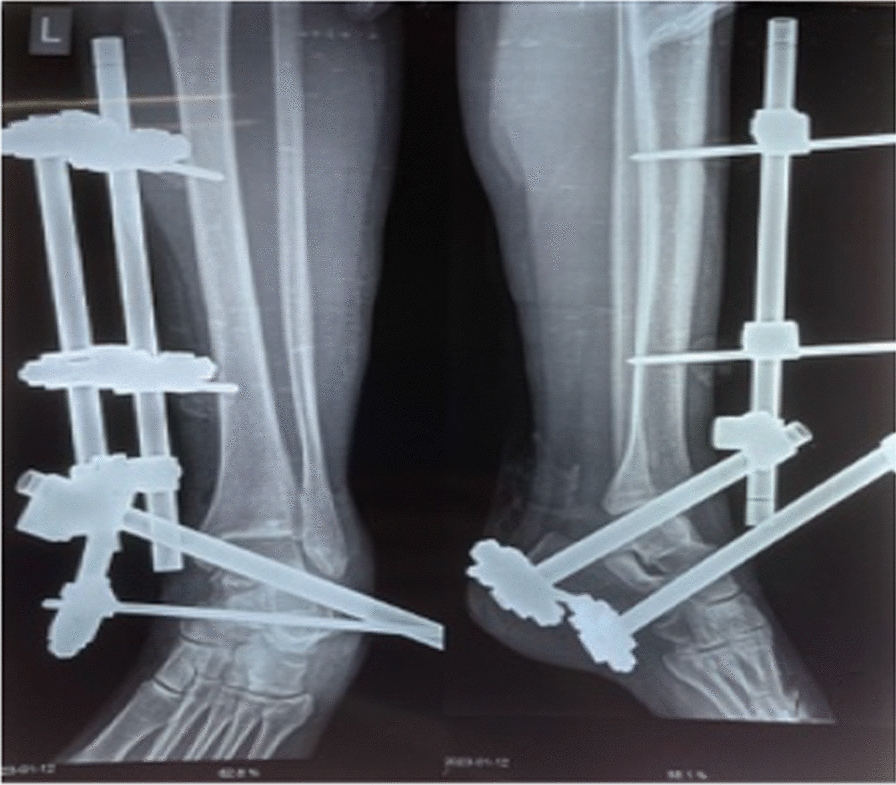
Radiograph post operation showing reduction of talus with external fixator in place

**Fig.4:**
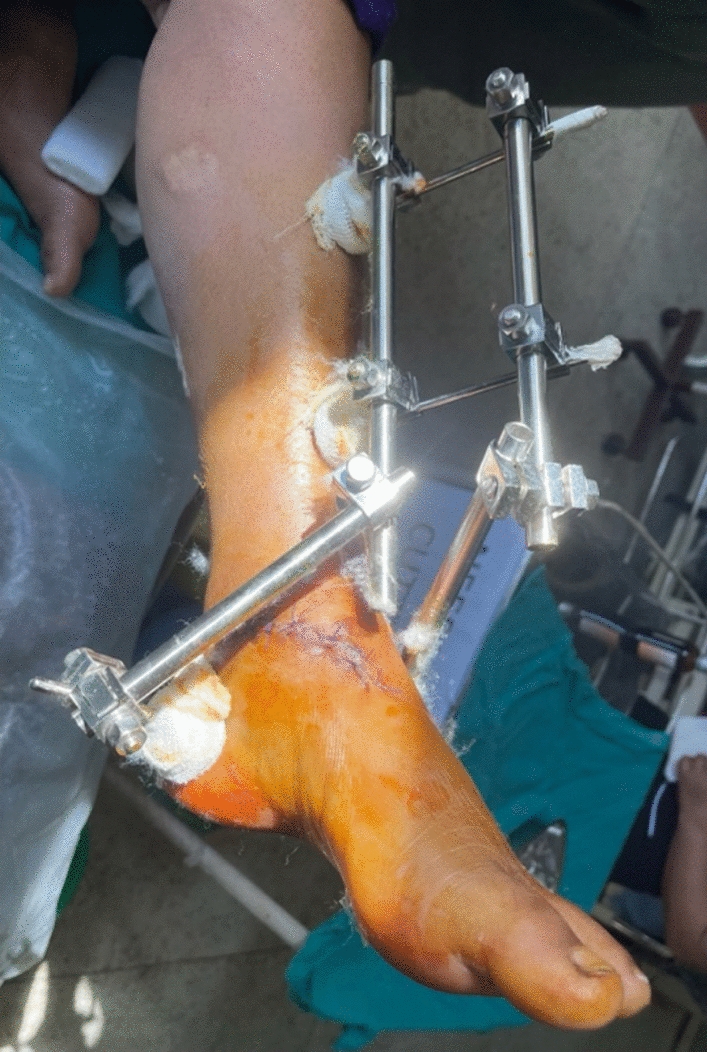
Clinical picture showing healthy wound at 2 weeks

**Fig.5:**
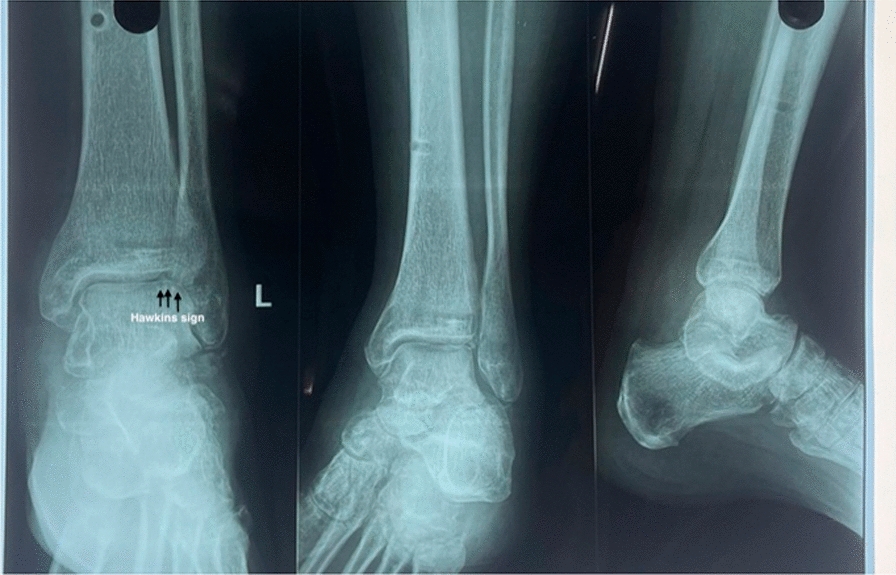
Radiograph 3 months post injury

After preoperative investigations, she was taken to the operating theater and further wound debridement and joint wash out was done about 6 hours after injury. The talus was attached with small strand of soft tissue only. Direct reduction was difficult, so was done with help of calcaneal Denham pin (for traction) without expanding the traumatic wound but the margin was freshened. Postreduction, no clinical nor radiological instability was observed. The range of motion was full. Distal pulses and capillary refill were normal after reduction. The ankle was stabilized with delta frame external fixator (Fig. 3). The wound was primarily closed with sutures and the patient was admitted in the ward for intravenous antibiotics and wound care and later discharged on the fourth postoperative day with toe-touch weight bearing on axillary crutches. She was reviewed at 2 weeks for suture removal. The traumatic wound healed uneventfully without evidence of superficial or deep infection (Fig. 4). Repeated radiographs at 6 weeks, 3 months, and 6 months posttrauma showed no evidence of avascular necrosis of the talus (Fig. 5). The external fixator was removed 6 weeks postoperatively and physiotherapy commenced. The patient has since been able to bear weight on the affected ankle with minimal tolerable pain and normal range of motion (ROM) and is on long-term follow up-in anticipation of posttraumatic arthritis or other sequelae.

## Discussion

**Fig.6:**
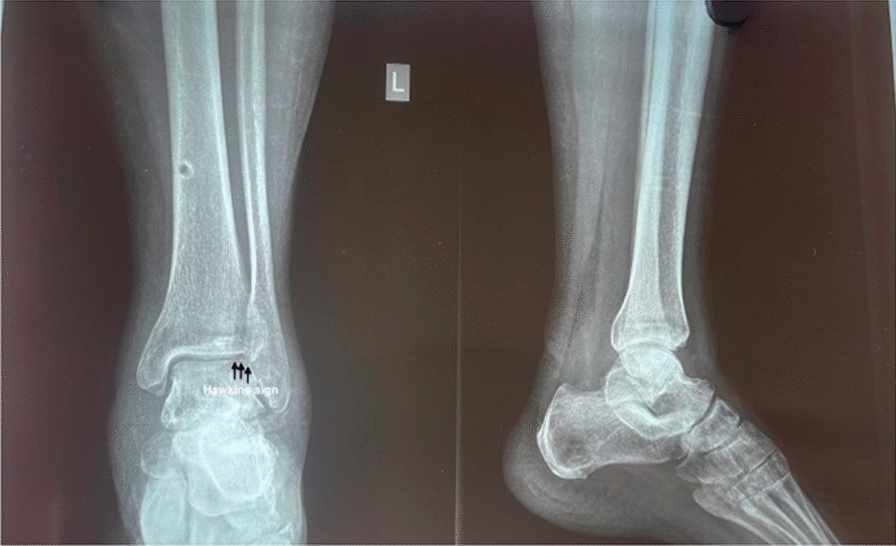
Radiograph 6 months post injury

Total talar dislocations are rare injuries with significant morbidity and disastrous complications for patients [6]. Due to the high-energy nature of these injuries and the vulnerable blood supply of the talus, AVN is commonly seen following these injuries [6]. Outcomes following total talar dislocations have shown high rates of AVN, up to 88%, with rates of talar dome collapse of approximately 28% of cases [6]. Posttraumatic osteoarthritis is also a significant complication, with rates ranging from 28% to 44% [6]. The most common complications affecting the prognosis are AVN and infection. In our case, the wound healed without any infection and follow-up x-ray shows the presence of Hawkins sign (Fig. 5 and Fig. 6) signifying absence of AVN. Also, the patient is on long-term follow-up for complications such as posttraumatic arthritis, which is still possible and may present years after initial injury. The management mentioned in our case resulted in an environment similar to the original biological state encouraging early revascularization. So, AVN was also avoided in our case as there was soft tissue, although minimal, and was still attached to the talus promoting revascularization [7], although the chances of AVN were high.

Many studies reported difficulty in reduction of the talus similar to our study, which is disagreed by Mitchell [8] who found that the reduction was easy. Similar to our method of using calcaneal pin, Newcomb and Brav [7] recommended adding a calcaneal pin and proximal tibial pin to help in the reduction.

Only a few case reports have been published about this injury. Kenwright and Taylor reported only two total talus dislocations out of 58 major talar injuries (3%) [9]. Furthermore, to our knowledge, this is probably the first case reporting done in our country about total talus dislocation. Published evidence-based treatment protocol for such injuries are fewer but treatment recommendations have evolved over the years from talectomy with tibio-calcaneal fusion to recent studies suggesting wound debridement, early reduction, and fixation(stabilization) to yield good clinical and functional outcomes, and our management is in accordance with recent case reviews.

There are different methods of stabilizing the talus and ankle joint after reduction of the talus. Methods include cast application, pinning with k wires, pinning along with back slab, and external fixators [10–12]. A delta frame external fixator stabilization was done in our study, similar to the approach by Tan *et al*. in a case of open talus dislocation [13].

## Conclusions

Open total talus dislocations are rare but serious injuries of the talus. Early diagnosis and immediate reduction, stabilization with proper debridement of wound and joint space prevents infection and chances of AVN of talus.

## Data Availability

Not applicable.

## Abbreviations

AVN: Avascular necrosis
ROM: Range of motion
